# Mechanizing the Laparoscope Operator in Sleeve Gastrectomy: A Single-Surgeon Before-and-After Study of a Pneumatic Endoscope-Holding Device (Lock-Arm®)

**DOI:** 10.1007/s11695-026-08737-8

**Published:** 2026-05-14

**Authors:** Susumu Inamine, Yasunori Uesato

**Affiliations:** Center for Metabolic and Bariatric Surgery, Ohama Daiichi Hospital, Naha, Japan

**Keywords:** Laparoscopic sleeve gastrectomy, Endoscope-holding device, Gastrointestinal surgeon shortage, Operative efficiency, Intraoperative hypothermia, Bariatric surgery

## Abstract

**Background:**

Japan faces a critical shortage of gastrointestinal surgeons. In the Japanese standard of care for laparoscopic surgery, three physicians are required: a primary surgeon, a first assistant, and a dedicated laparoscope operator. Freeing the third team member by using a mechanical endoscope-holding device could preserve surgical capacity, but its impact on operative efficiency and safety has not been rigorously quantified.

**Methods:**

We conducted a retrospective before-and-after study of 138 consecutive laparoscopic sleeve gastrectomy (LSG) cases performed by a single surgeon at a community bariatric center. The H group (*n* = 69) used a human laparoscope operator (Reverse Trendelenburg position, legs apart); the M group (*n* = 69) used the Lock-Arm® pneumatic passive endoscope holder (Reverse Trendelenburg position, legs together). Primary outcomes were OR occupancy time and operative time. Secondary outcomes included blood loss, intraoperative body temperature at three time points, Clavien-Dindo ≥ III complications, and postoperative length of stay. The study was reported in accordance with the STROBE statement.

**Results:**

Patient demographics were comparable between groups. OR occupancy time was shorter in the M group (175.7 ± 19.8 vs. 182.7 ± 21.2 min; MD − 7.0 min, 95% CI − 13.8 to − 0.1; *p* = 0.049). Operative time was significantly reduced (104.7 ± 14.5 vs. 117.1 ± 19.8 min; MD − 12.4 min, 95% CI − 18.2 to − 6.6; *p* < 0.0001; Cohen’s d = 0.72). Body temperature at end of surgery was higher in the M group (37.1 ± 0.4 vs. 36.6 ± 0.4 °C; MD + 0.43 °C, 95% CI + 0.29 to + 0.57; *p* < 0.0001; Cohen’s d = 1.04). No Clavien-Dindo ≥ III complications occurred in either group.

**Conclusions:**

Mechanizing the laparoscope operator role with the Lock-Arm® was associated with a significant reduction in operative time of approximately 12 min (10.6%), improved intraoperative thermoregulation, and maintained safety. This low-technology pneumatic device offers a practical strategy for sustaining laparoscopic surgical quality under surgical workforce constraints.

## Key Points

Introduction of a pneumatic passive endoscope holder (Lock-Arm®) in laparoscopic sleeve gastrectomy reduced operative time by 10.6% (12.4 min) without compromising safety.

The closed-leg Reverse Trendelenburg position enabled by the device significantly improved intraoperative thermoregulation (MD + 0.43 °C; Cohen’s d = 1.04), the largest effect size observed.

Eliminating the legs-apart lithotomy configuration reduces positioning-related risks — including lower extremity neuropathy and compartment syndrome — particularly relevant in patients with morbid obesity.

This low-cost, electricity-free device offers a practical strategy for sustaining laparoscopic surgical quality under workforce constraints without requiring robotic infrastructure.

## Introduction

Japan faces a critical and accelerating shortage of gastrointestinal surgeons. The number of gastrointestinal and general surgeons declined by more than 20% from 2002 to 2022 — the only major specialty to experience an absolute decline — while the total physician workforce grew by over 30% [1]. The Japan Society of Gastroenterological Surgery projects that the number of active surgeons under age 65 will fall to half the current level within 20 years [1].

In Japan, laparoscopic surgery conventionally requires three physicians: a primary surgeon, a first assistant, and a dedicated laparoscope operator. This differs from many Western bariatric centers where a single assistant handles both camera and instrument, halving physician demand. The Japanese three-physician model creates a staffing bottleneck increasingly difficult to sustain.

Task-shifting the camera operator role to non-physician personnel has been proposed but faces regulatory and cultural barriers. An alternative is to eliminate the human camera operator entirely with a passive mechanical endoscope-holding device. Robotic camera systems such as EndoAssist and AutoLap™ have demonstrated efficacy in randomized controlled trials [2, 3], but carry substantial cost and depend on electrical power and software infrastructure, limiting adoption in community settings.

The Lock-Arm® (System JP Co., Ltd., Hamamatsu, Shizuoka, Japan) is a passive, pneumatically actuated endoscope holder that mounts on the operating table rail. It is held rigid by compressed air and released instantaneously via a foot pedal, requiring no electricity, motors, or software — making it substantially less expensive than robotic alternatives and suitable for community hospitals. Our institution adopted this device to perform standardized LSG with a reduced physician team.

Prior to adoption, we anticipated that transferring camera repositioning responsibility from a dedicated human operator to the primary surgeon would increase operative time, since the surgeon must periodically pause dissection to release, reposition, and relock the arm. Contrary to this expectation, our clinical observation suggested that operative time decreased. A secondary, unanticipated observation was that the Lock-Arm® enables the patient to be positioned in the Reverse Trendelenburg position with legs together, rather than the legs-apart configuration required when a human assistant stands between the legs, with potential thermoregulatory benefits.

The aim of this study was to evaluate the impact of Lock-Arm® adoption on operative time and OR occupancy time as primary outcomes, and on intraoperative thermoregulation as a pre-specified secondary outcome — since the closed-leg position was anticipated to reduce heat loss by enabling full-body warming blanket coverage. Additional secondary outcomes included blood loss and perioperative complications.

## Methods

### Study Design and Reporting

This was a retrospective, single-center, single-surgeon before-and-after cohort study. The study was conducted and reported in accordance with the STROBE statement for observational studies [4]. Ethical approval was obtained from the Institutional Review Board of Ohama Daiichi Hospital (approval number: 276), and all procedures were performed in accordance with the Declaration of Helsinki. All patients provided written informed consent for surgery. Data were fully anonymized before analysis.

### Participants

Consecutive patients who underwent LSG performed by a single attending surgeon at the Ohama Daiichi Hospital, between January 2019 and December 2020 were eligible. No patients were excluded on clinical grounds. The cohort was divided chronologically: the H group (*n* = 69) comprised consecutive cases before Lock-Arm® adoption, in which a resident or surgical assistant manually controlled the laparoscope; the M group (*n* = 69) comprised the subsequent consecutive cases after Lock-Arm® adoption. All 138 cases are included without exclusion.

### Operative Technique

All procedures followed a standardized technique established by the operating surgeon before the study period. In both groups, patients were placed in the Reverse Trendelenburg position. In the H group, the legs were positioned apart (lithotomy configuration with stirrups) to allow the laparoscope assistant to stand between the patient’s legs and manually operate the camera throughout the procedure.

**Fig. 1:**
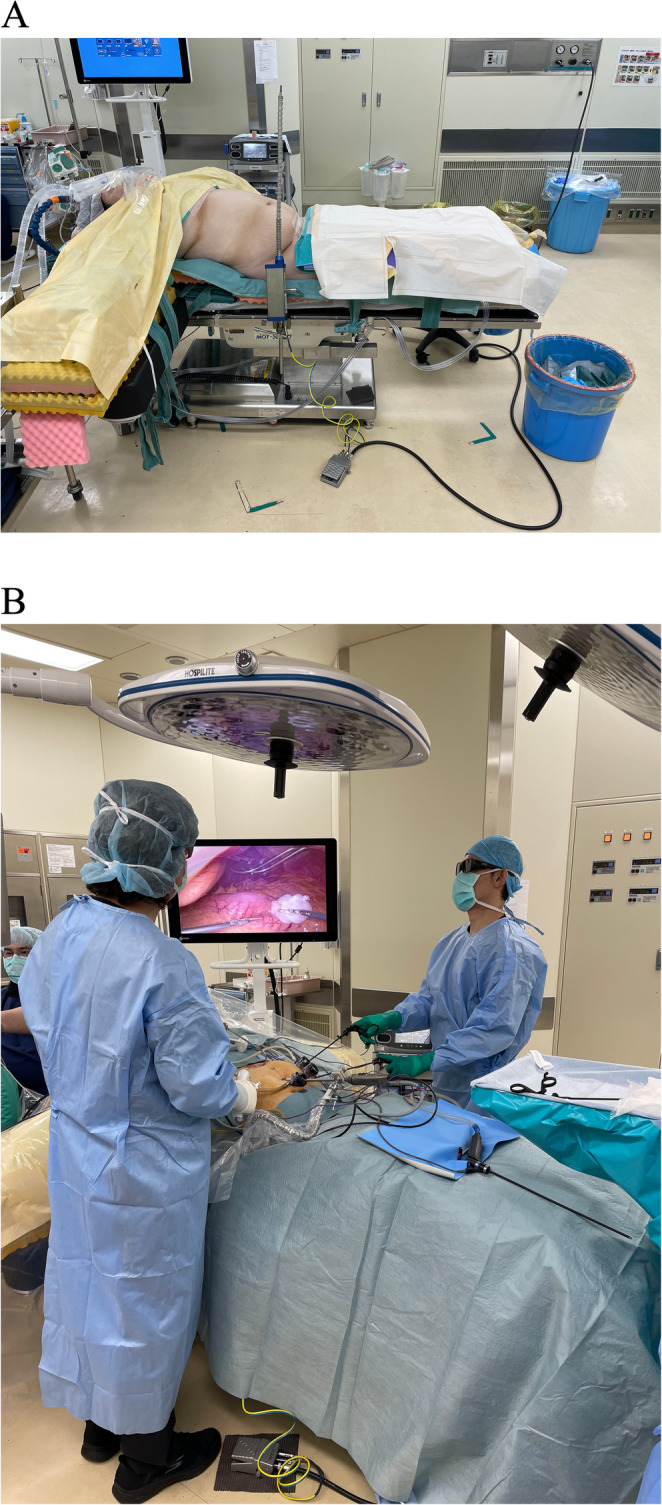
The Lock-Arm® pneumatic endoscope-holding device (System JP Co., Ltd., Hamamatsu, Shizuoka, Japan) in clinical use during laparoscopic sleeve gastrectomy. **A** Pre-operative setup in the closed-leg Reverse Trendelenburg position. The Lock-Arm® is mounted on the operating table rail. The pneumatic foot pedal (foreground) controls instantaneous fixation and release; the compressed air tube (yellow) connects to the hospital air supply. The closed-leg position allows full-body warming blanket coverage. **B** Intraoperative view showing the two-surgeon configuration. The primary surgeon (left) and first assistant (right) each operate with both hands simultaneously. The foot pedal (foreground) allows the surgeon to reposition the laparoscope without interrupting operative flow

**Fig. 2:**
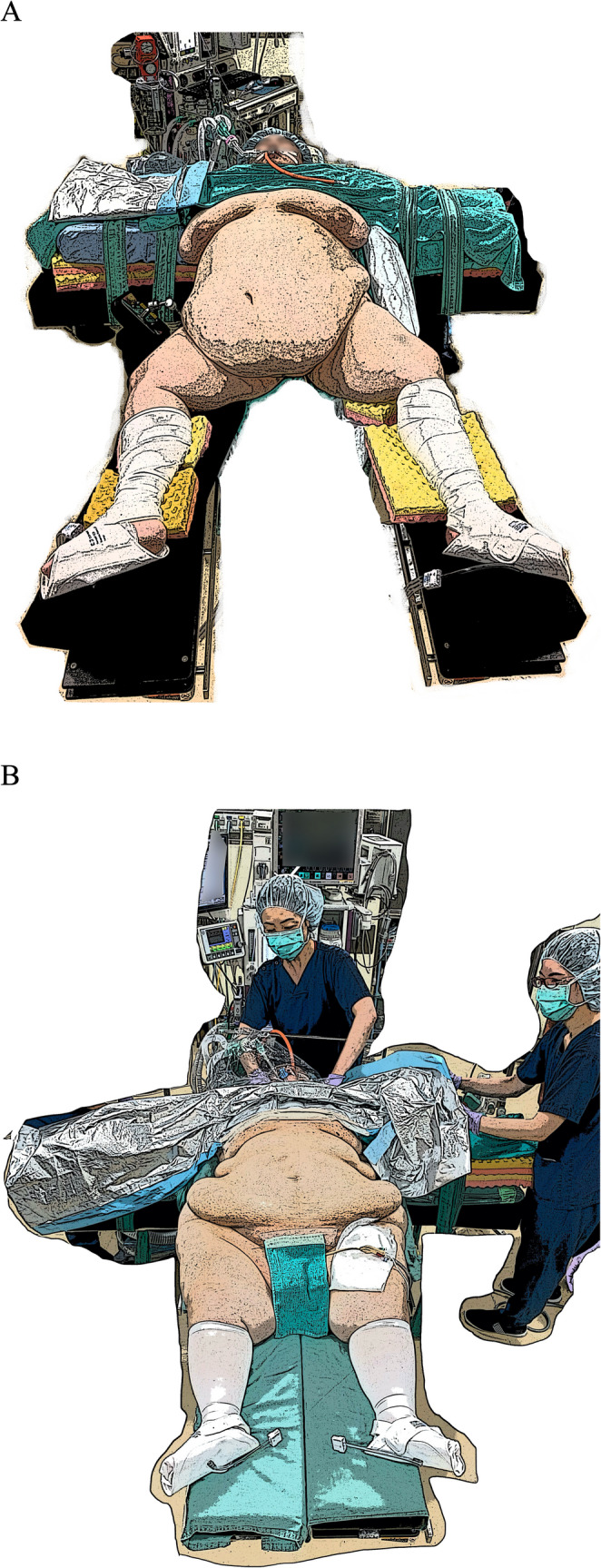
Patient positioning for laparoscopic sleeve gastrectomy. **A** H group: legs-apart Reverse Trendelenburg position with stirrups, used when a human laparoscope assistant stands between the patient’s legs. Note the exposed lower extremities, which limit warming blanket coverage. **B** M group: closed-leg Reverse Trendelenburg position. The photograph shows the position prior to draping; after positioning, a forced-air warming blanket is applied to cover the entire body surface including both lower extremities, which was not feasible in the legs-apart configuration of the H group

In the M group, the legs were kept together (closed-leg Reverse Trendelenburg position), as the Lock-Arm®, mounted on the ipsilateral operating table rail, replaces the need for an assistant between the legs (Fig. 1). The surgeon set the laparoscope angle at the start of each operative step, locked the arm via foot pedal, performed the step with both hands, then released and repositioned as needed. The pneumatic release mechanism allowed instantaneous transition between fixed and compliant states. Active warming with a forced-air warming blanket was applied in both groups; in the M group, the closed-leg position allowed the blanket to cover the full body surface including the lower extremities, which was not possible in the legs-apart configuration (Fig. 2).

### Outcomes

The primary outcomes were (1) OR occupancy time, defined as time from patient entry to exit from the operating room (minutes), and (2) operative time, defined as time from skin incision to skin closure (minutes). Secondary outcomes were intraoperative blood loss (g), body temperature at three time points (on OR arrival, at surgical start, and at surgical end), incidence of Clavien-Dindo grade ≥ III complications, and postoperative length of hospital stay.

### Statistical Analysis

Continuous variables are reported as mean ± standard deviation (SD). Between-group comparisons used the independent-samples t-test; categorical variables used the chi-squared test. For each outcome we report the mean difference (MD, M minus H), its 95% confidence interval (CI), and the p-value. Effect sizes for primary continuous outcomes are reported as Cohen’s d. A two-tailed *p* < 0.05 was considered statistically significant. Analyses were performed using Python 3.11 (scipy.stats v1.11, NumPy v1.24).

## Results

### Patient Demographics

The two groups were well-matched at baseline (Table 1). Mean age, body weight, BMI, and sex ratio were all comparable between groups with no statistically significant differences. Mean BMI was 43.0 ± 8.9 kg/m² (H group) and 42.7 ± 9.8 kg/m² (M group). All cases were performed using an identical standardized technique by the same surgeon.

**Table 1:** Patient demographics

Variable	H group (*n* = 69)	M group (*n* = 69)	*p* value
Age (years)	44.8 ± 10.8	46.3 ± 9.6	0.41
Body weight (kg)	113.3 ± 28.4	114.3 ± 26.5	0.83
BMI (kg/m²)	43.0 ± 8.9	42.7 ± 9.8	0.84
Sex (female/male)	41 / 28	47 / 22	0.38

Values are mean ± SD or n. BMI body mass index. *p*-values from independent-samples t-test or chi-squared test

### Operative Outcomes

Results are presented in Table 2. OR occupancy time was significantly shorter in the M group (175.7 ± 19.8 vs. 182.7 ± 21.2 min; MD − 7.0 min, 95% CI − 13.8 to − 0.1; *p* = 0.049; Cohen’s d = 0.34).

**Table 2:** Operative outcomes

Outcome	H group (*n* = 69)	M group (*n* = 69)	*p*	MD (95% CI)
OR occupancy time (min)	182.7 ± 21.2	175.7 ± 19.8	**0.049**	**−7.0 (− 13.8 to − 0.1)**
Operative time (min)	117.1 ± 19.8	104.7 ± 14.5	**< 0.0001**	**−12.4 (− 18.2 to − 6.6)**
Blood loss (g)	3.6 ± 7.5	2.9 ± 4.4	0.56	−0.6 (− 2.7 to + 1.4)
Body temp on arrival (°C)	36.1 ± 0.4	36.1 ± 0.5	0.86	+ 0.01 (− 0.14 to + 0.16)
Body temp at op start (°C)	36.6 ± 0.3	36.5 ± 0.3	0.47	−0.04 (− 0.14 to + 0.07)
Body temp at op end (°C)	36.6 ± 0.4	37.1 ± 0.4	**< 0.0001**	**+ 0.43 (+ 0.29 to + 0.57)**
Clavien-Dindo ≥ III	0 / 69 (0%)	0 / 69 (0%)	—	—
Postoperative stay (days)	No significant difference		NS	—

Values are mean ± SD or n (%). MD mean difference (M minus H); CI confidence interval; NS not significant; OR operating room. Bold *p*-values and MD indicate statistically significant differences. Positive MD = higher in M group; negative MD = lower in M group

Operative time was markedly shorter in the M group (104.7 ± 14.5 vs. 117.1 ± 19.8 min; MD − 12.4 min, 95% CI − 18.2 to − 6.6; *p* < 0.0001; Cohen’s d = 0.72), a 10.6% reduction in mean operative duration. The standard deviation of operative time was notably narrower in the M group (14.5 vs. 19.8 min), suggesting greater procedural consistency.

Intraoperative blood loss did not differ significantly (2.9 ± 4.4 vs. 3.6 ± 7.5 g; MD − 0.6 g, 95% CI − 2.7 to + 1.4; *p* = 0.56). Body temperature on OR arrival and at the start of surgery were similar between groups (*p* = 0.86 and *p* = 0.47, respectively). Body temperature at the end of surgery was significantly higher in the M group (37.1 ± 0.4 vs. 36.6 ± 0.4 °C; MD + 0.43 °C, 95% CI + 0.29 to + 0.57; *p* < 0.0001; Cohen’s d = 1.04). No Clavien-Dindo grade ≥ III complications occurred in either group. Postoperative length of stay was not significantly different.

## Discussion

The primary finding of this study is that replacing a human laparoscope operator with the Lock-Arm® pneumatic endoscope holder in LSG was associated with a significant reduction in operative time of approximately 12 min (10.6%, Cohen’s d = 0.72) and a reduction in OR occupancy time of 7 min, without any increase in blood loss or complications. Crucially, these improvements were achieved while simultaneously reducing physician staffing requirements from three to two per case, directly addressing the workforce constraints described in the Introduction.

The operative time reduction was contrary to our preoperative hypothesis. The most plausible explanation relates to image stability: when a human assistant holds the laparoscope, subtle drift and suboptimal framing are unavoidable, whereas the Lock-Arm® fixes the visual field mechanically. The fixed field also compels a more compact operative technique, as all maneuvers must be performed within the current field of view. While this insight is based on the primary surgeon’s experience and cannot be objectively quantified, it is consistent with the 10.6% reduction in operative time and the narrower SD in the M group (14.5 vs. 19.8 min), suggesting greater procedural consistency.

The improvement in end-of-surgery body temperature (MD + 0.43 °C, Cohen’s d = 1.04) is attributable to the change in leg position. The legs-apart configuration required in the H group exposed the lower extremities and precluded full warming blanket coverage; the closed-leg position in the M group allowed full-body coverage, reducing heat loss from the lower limbs. The effect size (Cohen’s d = 1.04) was the largest of any outcome in this study. Perioperative hypothermia is associated with increased risk of cardiac events [5], impaired coagulation [6], and surgical site infections — risks particularly relevant in patients with morbid obesity, in whom hypothermia has been documented even with active warming [7].

The 7.0-minute reduction in OR occupancy time warrants interpretation. Since operative time was reduced by 12.4 min while OR occupancy time was reduced by only 7.0 min, non-operative time was approximately 5 min longer in the M group. The reasons are not fully explained by the available data; Lock-Arm® setup is performed in parallel with anesthesia induction and adds negligible time clinically. Variability in anesthesia and nursing workflow may have contributed. Regardless, OR occupancy reduction was driven entirely by the improvement in operative time, representing a meaningful efficiency gain in high-volume programs.

From a workforce perspective, the Lock-Arm® enabled LSG with one attending surgeon, one part-time assistant, and a clinical engineer — directly addressing the Japanese three-physician staffing bottleneck. Its pneumatic mechanism requires no electrical power, no software, and is substantially less expensive than robotic camera systems, making it immediately implementable at community bariatric centers.

The closed-leg position also reduces positioning-related risks associated with the legs-apart lithotomy configuration. Lower extremity neuropathy occurs in approximately 1.5% of patients in the lithotomy position, with operative duration and high BMI as primary risk factors [8, 9]. Compartment syndrome, though rare, is catastrophic and preferentially occurs in prolonged, high-BMI cases [9]. Pressure mapping studies have demonstrated higher peak pressures at bony prominences in the lithotomy versus closed-leg supine position [10]. Although these parameters were not directly measured in the present study, no positioning-related complications occurred in either group, and the M group benefited from the inherently lower-risk posture throughout.

Several limitations must be considered. First, the non-randomized before-and-after design introduces the risk of secular trend bias. However, the primary surgeon had already performed more than 500 LSG procedures before the study period began and had established a fully standardized technique; a pure learning-curve explanation for the 12-minute improvement is therefore unlikely. The narrower SD of operative time in the M group further supports a mechanistic rather than experiential explanation. Second, surgeon-reported qualitative experiences — such as the compaction of operative movements within the fixed camera field — cannot be objectively quantified from the available data, and their contribution to the observed time reduction remains inferential. Third, patient comfort in the closed-leg Reverse Trendelenburg position, joint stress considerations, and assistant ergonomics were not formally assessed. Fourth, the sample of 69 per group limits power to detect rare events. Prospective multicenter studies are warranted.

## Conclusions

In this single-surgeon before-and-after study of 138 LSG cases, introduction of the Lock-Arm® was associated with a 10.6% reduction in operative time, reduced OR occupancy, and clinically meaningful improvement in intraoperative thermoregulation, without adverse impact on safety. The closed-leg position simultaneously avoids lithotomy-related risks — including lower extremity neuropathy, compartment syndrome, and elevated skin interface pressures — of particular concern in patients with morbid obesity. This low-cost, electricity-free device offers multiple concurrent benefits: improved operative efficiency, better thermoregulation, reduced positioning-related risk, and reduced physician staffing requirements.

## Data Availability

The datasets generated and analyzed during the current study are not publicly available due to patient privacy and institutional regulations, but are available from the corresponding author on reasonable request.
